# Prevalence of healthcare-associated infections and antimicrobial use among inpatients in a tertiary hospital in Fiji: a point prevalence survey

**DOI:** 10.1186/s13756-020-00807-5

**Published:** 2020-08-28

**Authors:** M. J. Loftus, S. J. Curtis, R. Naidu, A. C. Cheng, A. W. J. Jenney, B. G. Mitchell, P. L. Russo, E. Rafai, A. Y. Peleg, A. J. Stewardson

**Affiliations:** 1 Department of Infectious Diseases, The Alfred Hospital and Central Clinical School, Monash University, Melbourne, Australia; 2 Colonial War Memorial Hospital, Suva, Fiji; 3 School of Public Health and Preventive Medicine, Monash University, Melbourne, Australia; 4 Fiji National University, Suva, Fiji; 5 School of Nursing and Midwifery, University of Newcastle, Callaghan, Australia; 6 Department of Nursing Research, Cabrini Institute, Malvern, Australia; 7 Department of Nursing and Midwifery, Monash University, Frankston, Australia; 8 Fiji Ministry of Health and Medical Services, Suva, Fiji; 9 Infection and Immunity Program, Monash Biomedicine Discovery Institute, Department of Microbiology, Monash University, Clayton, Australia

**Keywords:** Healthcare associated infection, Antimicrobial use, Infection prevention, Surveillance, Point prevalence study, Antimicrobial stewardship, Fiji

## Abstract

**Background:**

Healthcare-associated infections (HAIs) and antimicrobial use (AMU) are important drivers of antimicrobial resistance, yet there is minimal data from the Pacific region. We sought to determine the point prevalence of HAIs and AMU at Fiji’s largest hospital, the Colonial War Memorial Hospital (CWMH) in Suva. A secondary aim was to evaluate the performance of European Centre for Diseases Prevention and Control (ECDC) HAI criteria in a resource-limited setting.

**Methods:**

We conducted a point prevalence survey of HAIs and AMU at CWMH in October 2019. Survey methodology was adapted from the ECDC protocol. To evaluate the suitability of ECDC HAI criteria in our setting, we augmented the survey to identify patients with a clinician diagnosis of a HAI where diagnostic testing criteria were not met. We also assessed infection prevention and control (IPC) infrastructure on each ward.

**Results:**

We surveyed 343 patients, with median (interquartile range) age 30 years (16–53), predominantly admitted under obstetrics/gynaecology (94, 27.4%) or paediatrics (83, 24.2%). Thirty patients had one or more HAIs, a point prevalence of 8.7% (95% CI 6.0% to 12.3%). The most common HAIs were surgical site infections (*n* = 13), skin and soft tissue infections (7) and neonatal clinical sepsis (6). Two additional patients were identified with physician-diagnosed HAIs that failed to meet ECDC criteria due to insufficient investigations. 206 (60.1%) patients were receiving at least one antimicrobial. Of the 325 antimicrobial prescriptions, the most common agents were ampicillin (58/325, 17.8%), cloxacillin (55/325, 16.9%) and metronidazole (53/325, 16.3%). Use of broad-spectrum agents such as piperacillin/tazobactam (*n* = 6) and meropenem (1) was low. The majority of prescriptions for surgical prophylaxis were for more than 1 day (45/76, 59.2%). Although the number of handwashing basins throughout the hospital exceeded World Health Organization recommendations, availability of alcohol-based handrub was limited and most concentrated within high-risk wards.

**Conclusions:**

The prevalence of HAIs in Fiji was similar to neighbouring high-income countries, but may have been reduced by the high proportion of paediatric and obstetrics patients, or by lower rates of inpatient investigations. AMU was very high, with duration of surgical prophylaxis an important target for future antimicrobial stewardship initiatives.

## Background

Healthcare associated infections (HAIs) represent a significant burden of disease globally, contributing to increased morbidity, mortality and healthcare costs [1–3]. However many HAIs are preventable, and certain initiatives such as environmental cleaning bundles [4], hand hygiene [5] or guidelines for the insertion and management of central venous catheters [6] have managed to reduce the rates of selected HAIs, including within low- and middle-income settings [7].

An accompanying and related threat is that of antimicrobial resistance, which is promoted by antimicrobial use (AMU) and tends to concentrate in hospital settings where use of these agents is most intense [8–10]. Previous studies have shown that up to one-third of AMU in hospitals is unnecessary or inappropriate [11–14]. HAIs can act as a key driver of antimicrobial resistance by both increasing the overall demand for antimicrobials, as well as the likelihood of patients acquiring a drug-resistant pathogen during their hospital stay.

To select and prioritise suitable interventions to prevent both HAIs and inappropriate AMU, quality surveillance data is needed. Continuous, prospective surveillance can provide detailed information, however demands the most resources and is impractical in many settings. Use of a point prevalence survey (PPS), especially if repeated over time, is a pragmatic alternative that is far less resource-intensive. One of the most commonly used standardised PPS protocols to assess HAIs and AMU was developed by the European Centre for Disease Prevention and Control (ECDC) [15]. Although originally designed for use in the predominantly high-income setting of Europe [16, 17], it has subsequently been employed by many other countries globally [18–20], including within low- and middle-income country (LMIC) settings [21–24].

No estimates of the prevalence of HAIs within the Pacific Islands region have ever been published, nor point prevalence data of hospital AMU. We sought to address this knowledge gap by conducing a PPS of both HAIs and AMU at Fiji’s largest hospital. Our objectives were to: estimate the prevalence of HAIs and describe their distribution by type and patient group, outline the prevalence of AMU and the most commonly used antimicrobials, and describe key infection prevention and control (IPC) infrastructure present at the hospital. We also sought to assess the suitability of ECDC HAI criteria in the setting of Fiji, a middle-income country.

## Methods

### Setting

We conducted a PPS of HAIs and AMU at the Colonial War Memorial Hospital (CWMH), Suva, over five consecutive weekdays in October 2019. CWMH is a 500-bed tertiary hospital and the largest healthcare facility in Fiji, serving a local catchment population of over 400,000 people while also acting as the national referral centre for Fiji’s other divisional hospitals in Labasa (175 beds) and Latouka (275 beds). It has separate adult, paediatric and neonatal intensive care units (ICUs), and offers specialty services including cardiology, orthopaedics, plastic surgery, urology and neurosurgery. CWMH manages over 22,000 admissions per year, it is predominantly a public facility however does contain one private inpatient ward. Presently there is no routine screening of CWMH inpatients for carriage of resistant pathogens.

### Study design

We used a modified version of the 2016 ECDC methodology [15]. All changes from the original ECDC PPS are outlined in Additional file 1: Table S1.

ECDC criteria to diagnose HAIs frequently include results of microbiology and radiology investigations, which are performed less often in low- and middle-income countries [25]. We therefore included survey questions to identify instances when ECDC criteria had not been met due to key investigations not being performed, but physicians had made a clinical diagnosis of HAI. This was to help avoid an underestimation of HAIs, and also to assess the performance of ECDC criteria in our setting. After consultation with CWMH staff (RN, AJ), these questions were targeted to skin and soft tissue infection, pneumonia and *Clostridioides difficile* infection due to their anticipated frequency, the reliance on investigations to meet ECDC criteria, and the likelihood that these same investigations may not be performed at CWMH. In the case of *C. difficile* infections, no laboratory testing is available at all. These additional questions are outlined in Additional file 1: Table S2.

### Ward selection

All acute care inpatient wards at CWMH were included in the study, with the exception of the psychiatry ward and the emergency department.

### Patient selection

We included all patients admitted on a study ward prior to 8 am on the day of the survey, who had not been discharged from the ward by the time the survey was conducted. Patients absent from the ward at the time of the survey were excluded.

### Data collection and definitions

The data collection team consisted of two visiting survey leaders (one Infectious Diseases physician [ML] and one Research Assistant who performed data collection in the recent Australian PPS [SC] [19]), and four local IPC staff (AD, AP, AS, EM). Immediately prior to the PPS, these local staff received two full days of training in HAI surveillance, data collection methodology, and use of the survey tools. Each day, research pairs were formed consisting of one visiting and one local data collector. Pairings were sequentially rotated every half-day to maximise standardisation of data entry. This team structure was designed to increase the capacity of local IPC staff to perform PPSs in the future.

Teams used mobile tablet devices to enter data using REDCap electronic data capture tools hosted at Monash University [26]. This data collection tool was adapted from those used in recent Singaporean [18] and Australian [19] studies, using branching logic based on ECDC HAI definitions to assist data collectors. Patients with either a fever within the last 24 h or current antimicrobial prescription underwent a full chart assessment, in other cases demographic data alone were collected.

Patient-level data including age, sex, medical specialty, length of stay, presence of invasive device, and details of antimicrobial use were all collected at the time of visit to the ward. Antimicrobial use and indication were determined by assessing drug charts and patient notes. Medical specialty was defined by the specialty of the admitting consultant. Wards were divided into high-risk (ICUs and the Burns ward) and low-risk (all others), with ICUs defined as wards with higher nurse:patient ratios and the capacity to provide inotropes and invasive ventilation. CWMH’s ‘Maternity ICU’ was therefore categorised as a low-risk ward for the purpose of this study, given it did not meet these criteria.

On the wards, teams had access to patient notes and drug charts (both paper-based) as well as plain radiograph films. Pathology, microbiology and selected radiology investigations (eg CT, MRI) could also be accessed on the hospital’s electronic database. After assessing this information, researchers determined whether ECDC HAI criteria had been met. Criteria for any given HAI had to be clearly documented by healthcare workers in the notes, or demonstrable from pathology, microbiology or radiology results. Unless all criteria were clearly met, a HAI was not considered to be present. In uncertain or contentious cases, the research teams would consult each other and reach consensus regarding interpretation of patient notes and the application to HAI criteria.

We also collected information about IPC infrastructure on each ward and use of transmission-based precautions. This included the number of alcohol-based handrub (ABHR) dispensers and sinks, the number of patient toilets and bathrooms, and the number and indication(s) for patients in transmission-based precautions.

For HAI and AMU, the number of patients included in the PPS was the denominator, while a ward’s maximum bed capacity was the denominator when assessing IPC infrastructure. When evaluating patient numbers, neonates sharing a bed with their mothers were included in the PPS if they continued to be reviewed daily by the paediatric team.

### Statistical analysis

All data were analysed using R version 3.6.2 [27]. The point prevalence of HAIs was estimated from the proportion of patients in the sample with at least one HAI. We computed confidence intervals for proportions using the Clopper-Pearson method.

### Ethical considerations

Approval, with a waiver of the requirement for individual consent, was provided by the Alfred Hospital Ethics Committee (400/19), the Fiji National Health Research Ethics Review Committee (2019.82.C.D), and the CWMH Medical Superintendent.

## Results

### Patient baseline characteristics

There were 355 eligible patients who were present when wards were assessed at 8 am and had not been discharged prior to the survey. Twelve were excluded as they were off the ward at the time of the survey, leaving 343 patients for the final analysis.

The median patient age was 30 years (IQR 16–53 years), 147 (42.9%) were male, and 238 (69.4%) had at least one invasive device present with the most common being a peripheral vascular catheter (63.6%, 218/343). The median hospital length of stay prior to conducting the PPS was 4 days (IQR 2–8 days, range 0–87 days). Further clinical and demographic attributes of HAI and no HAI groups are outlined in Table 1.

**Table 1:** Characteristics of patient with and without healthcare associated infection (HAI)

	All patients	No HAI	HAI
**Patients, n**	343	313	30
**Male, n (%)**	147 (42.9)	131 (41.9)	16 (53.3)
**Age, n (%)**			
Neonate	52 (15.2)	46 (14.7)	6 (20.0)
0–14	33 (9.6)	31 (9.9)	2 (6.7)
15–29	81 (23.6)	74 (23.6)	7 (23.3)
30–44	66 (19.2)	63 (20.1)	3 (10.0)
45–59	57 (16.6)	50 (16.0)	7 (23.3)
60+	54 (15.7)	49 (15.7)	5 (16.7)
**Medical specialty, n (%)**			
Medical	91 (26.5)	83 (26.5)	8 (26.7)
Surgical	75 (21.9)	67 (21.4)	8 (26.7)
Obstetrics & Gynaecology	94 (27.4)	88 (28.1)	6 (20.0)
Paediatrics	83 (24.2)	75 (24.0)	8 (26.7)
Paediatrics excluding NICU	69 (20.1)	66 (21.1)	3 (10.0)
NICU	14 (4.1)	9 (2.9)	5 (16.7)
**Ward, n (%)**			
ICU/Burns wards	32 (9.3)	23 (7.3)	9 (30.0)
Other	311 (90.7)	290 (92.7)	21 (70.0)
**LOS in days until PPS performed, median (IQR)**			
Overall	4 (2–8)	3 (2–7)	17.5 (6.5–22)
Neonates	4.5 (3–9)	3 (2–9)	8.5 (5–16)
ICU/Burns wards	8 (4–17)	6 (3.5–13)	18 (6–21)
**Devices, n (%)**			
Any invasive device	238 (69.4)	212 (67.7)	26 (86.7)
PVC	218 (63.6)	197 (62.9)	21 (70.0)
CVC	19 (5.5)	14 (4.5)	5 (16.7)
Urinary catheter	26 (7.6)	22 (7.0)	4 (13.3)
Invasive ventilation	4 (1.2)	4 (1.3)	0 (0.0)
**Documented fever in last 24 h, n (%)**	21 (6.1)	14 (4.5)	7 (23.3)
**Receiving antimicrobial therapy, n (%)**	206 (60.1)	176 (56.2)	30 (100.0)

ICU/Burns wards: adult ICU, paediatric ICU, neonatal ICU and Burns. Other wards: 17 wards with specialities including general medicine, cardiology, general surgery, obstetrics, gynaecology and paediatrics.

*LOS* Length of stay, *NICU* Neonatal intensive care unit, *PVC* Peripheral vascular catheter, *CVC* Central vascular catheter

### HAI prevalence

As 36 HAIs were detected in 30 patients, the overall prevalence of patients with one or more HAIs was 8.7% (95% CI 6.0–12.3%). The most common HAIs were surgical site infection (SSI) (*n* = 13, prevalence 4%), skin and soft-tissue infection (7, 2%) and neonatal clinical sepsis (6, 2%) (Table 2). Criteria for neonatal clinical sepsis was met by 12% (6/52) of all neonates and 36% (5/14) of those within the neonatal ICU.

**Table 2:** Healthcare associated infections (*n* = 36) among 30 patients

Surgical site infection	13 (36.1%)
Skin and soft tissue infection	7 (19.4%)
Neonatal clinical sepsis	6 (16.7%)
Pneumonia	4 (11.1%)
Urinary tract infection	2 (5.6%)
Bone and joint infection	1 (2.8%)
Bloodstream infection (PVC-related)	1 (2.8%)
Bloodstream infection	1 (2.8%)
Unspecified sepsis	1 (2.8%)

*PVC* Peripheral venous catheter

We identified two patients with HAIs diagnosed by treating physicians without meeting ECDC criteria. This was due to either the necessary microbiology (one case of skin and soft tissue infection) or radiology (one case of pneumonia) investigations not being performed.

### Organisms

Causative organisms were identified for 58% (21/36) of HAIs. Accounting for 4 patients with multiple HAIs caused by the same organism, there were 17 distinct organisms identified from 16 patients.

These organisms were *Staphylococcus aureus* (*n* = 6), *Klebsiella pneumoniae* (4), *Pseudomonas aeruginosa* (3), *Klebsiella oxytoca* (1), *Citrobacter freundii* (1), *Proteus mirabilis* and one unspecified Gram negative bacilli. Half (3/6) of the *S. aureus* isolates were methicillin-resistant, while 71% (5/7) of *Enterobacterales* were non-susceptible to third-generation cephalosporins. No isolates demonstrated non-susceptibility to carbapenems.

### Antimicrobial consumption

Overall, 60.1% (206/343) of patients were receiving at least one antimicrobial at the time of the survey. Drug charts for five patients were unavailable yet their medical notes clearly indicated ongoing receipt of antimicrobials. Among those with drug charts available, a total of 325 antimicrobials were being given to 201 patients; 102 patients were receiving one, 77 patients were receiving two, and 22 patients were receiving three or more agents.

The most commonly prescribed antimicrobials were ampicillin, cloxacillin, metronidazole and gentamicin (Table 3). There was minimal use of broad-spectrum agents such as piperacillin-tazobactam (*n* = 6) and meropenem (1), although the hospital was experiencing a piperacillin-tazobactam shortage at the time of the PPS.

**Table 3:** Antimicrobial use at Colonial War Memorial Hospital (*n* = 325 antimicrobials)

**Antimicrobial agent, n(%)**	
Ampicillin	58 (17.8%)
Cloxacillin	55 (16.9%)
Metronidazole	53 (16.3%)
Gentamicin	39 (12.0%)
Benzylpenicillin	31 (9.5%)
Ceftriaxone	17 (5.2%)
Other	72 (22.2%)
**Route of administration, n(%)**	
Parenteral	235 (72.3)
Enteral	89 (27.4)
Other	1 (0.3)
**Indication, n(%)**	
Treatment of community acquired infection	149 (45.8)
Surgical prophylaxis	76 (23.4)
0–1 day duration	31 (40.8)
> 1 day duration	45 (59.2)
Treatment of healthcare associated infection	53 (16.3)
Medical prophylaxis	23 (7.1)
Unknown	16 (4.9)
Other	8 (2.5)
**Length of antimicrobials prior to PPS, median (IQR)**	
Overall	2 days (1–4)
ICU/Burns wards	3 days (2–5)
Other wards	2 days (1–4)

The most common indication for antimicrobials was treatment of community acquired infection (149/325, 45.8%), followed by surgical prophylaxis (76/325, 23.4%) and treatment of healthcare associated infection (53/325, 16.3%).

Across all prescriptions, the median length of time from antimicrobial commencement to the PPS was 2 days (IQR 1–4 days, range 0–37 days). The 76 prescriptions for surgical prophylaxis shared the same median time of 2 days (IQR 1–3 days); only 40.8% (31/76) of surgical prophylaxis prescriptions had been given for 1 day or less.

### Infection prevention and control infrastructure

The 22 assessed wards had a maximum capacity of 457 beds, however many beds were not in use year-round. Excluding the private ward where 9 of 31 beds (29%) were in single rooms, for the remaining 426 beds there were only 6 single rooms (1.4%). Non-single rooms ranged in size from 2 to 10 beds.

There were 61 bathrooms containing 68 toilets. Thirteen bathrooms were designed for the use of a single patient, with the majority of these (9/13, 69%) being ensuites on the private ward. The remaining 55 toilets served 444 beds; an average of 8.1 patients per toilet. In one particular area, however, 46 patients shared one toilet and bathroom.

Across the surveyed wards, 3.2% (11/343) of patients were in transmission-based precautions at the time of the PPS. The most common indication was infection with a multi-resistant Gram-negative organism (5/11, 45%).

Hand hygiene resources were concentrated within the ICU/Burns wards, with an average of 1.0 ABHR dispensers per bed, compared to 0.15 ABHR dispensers per bed on non-ICU/Burns wards. All patients on transmission-based precautions – regardless of location – had ABHR present at the foot of their bed. Handwashing basins were more evenly distributed, with 2.7 handwashing basins per 10 beds on ICU/Burns wards, and 2.3 per 10 beds on other wards.

## Discussion

This is the first published point prevalence survey of HAIs and antimicrobial use from a Pacific Island country or territory. We found that 8.7% of inpatients had a HAI, with SSIs most commonly responsible. In addition, 60.1% of all CWMH inpatients were receiving at least one antimicrobial, with a high proportion of surgical prophylaxis extending for more than 1 day. A key component of our project was capacity building, providing training to local staff in both PPS methodology as well as the use of mobile technology for data entry. We found a tablet-based PPS to be an efficient means of obtaining and collating survey data within a LMIC setting.

The HAI prevalence from our research in Fiji is similar or slightly lower than estimates from other studies in LMICs which also used ECDC criteria, such as 8.2% in Ghana, 8.4% in Pakistan, 13.5% in Botswana and 11.5% across four Latin American countries [21–23]. Compared to high-income settings, Fiji’s HAI prevalence is higher than that reported in the USA (3.2%), Europe (6.5%), and Japan (7.4%) but lower than rates in Australia (9.9%) and Singapore (11.9%) [17–20, 28]. This relatively low HAI prevalence in Fiji is discordant with a previous systematic review suggesting HAIs were approximately twice as common in lower-income countries compared to higher-income countries, especially when HAI prevalence was assessed prospectively using standardised definitions [29]. There are a number of factors that may have reduced the prevalence of HAIs in this PPS. First, unlike many other hospitals in LMICs, CWMH has an IPC team consisting of a physician and five nurses, that can target the hospital’s limited IPC resources. For example, the ABHR dispensers were appropriately concentrated within high-risk areas, and availability of handwashing basins exceeded World Health Organization minimum recommendations of 1 sink: 10 beds [30]. Second, our PPS contained a very high proportion of paediatric (23.9%) and obstetrics/gynaecology (27.7%) patients, many of whom were otherwise healthy and only admitted peri-partum. These two groups have been shown to have lower HAI prevalence compared to other specialties [31]. With neonatal ICU patients excluded, the HAI prevalence among children in our study was only 4.4% (3/69). Other recent PPSs from LMICs also include similarly high proportions of paediatric (from 15 to 26%) [21–24] and obstetric (from 19 to 30%) [21, 23] patients, whereas recent studies from high-income countries showing higher HAI prevalence excluded paediatric patients [18, 19]. Third, inpatients in Fiji may not have been as sick as those in other studies, although no comorbidity data was collected to allow direct comparison. However, the use of invasive devices associated with the sickest patients was much lower at CWMH compared to high-income settings. Only 5.5% of patients at CWMH had central venous catheters compared to 7–16% in PPSs from high-income settings [16, 18–20, 28], for invasive ventilation this comparison was 1.2% at CWMH versus 2–5% elsewhere [16, 18, 19, 28]. Fourth, overall antimicrobial use in Fiji was high, including prolonged surgical prophylaxis. It is possible that some of these patients were being treated for a HAI without documentation of this intention in the notes.

A final potential factor that could lower the recorded HAI prevalence is reduced frequency of radiology or microbiology investigations. The low use of diagnostics in LMIC settings and its impact on HAI surveillance has been highlighted by previous authors [21]. We sought to directly address this in our study design, and detected a further two patients who may have met HAI criteria if not for missing radiology or microbiology. Including those patients would only slightly increase HAI prevalence from 8.7 to 9.3% (32/343). This suggests that ECDC criteria performed well in our setting, but other LMICs may want to consider modifications when performing PPSs, depending on the local availability and use of radiology and microbiology testing.

SSIs were the most frequent HAI in our study, causing over one-third of all HAIs. The problem of SSIs in LMICs has been noted in multiple previous HAI studies, where they were ranked either first or second [21–24, 32]. By contrast, SSIs routinely rank third or lower among HAIs in high-income settings, with the exception of Australia [19]. This discrepancy could reflect either suboptimal perioperative management practices or the relative ease of diagnosing SSIs compared to other HAIs; in the ECDC protocol a physician diagnosis criteria is included for all SSI categories. Another notable finding was the low prevalence of urinary tract infection HAIs at CWMH – only two were detected across the entire cohort – when these often feature among the top causes of HAIs in high-income settings [16, 19]. A likely contributor was the infrequent use of urinary catheters at CWMH: present in only 7.6% of patients compared with 18 to 26% in high-income countries [16, 18, 19, 28].

Six out of every 10 patients at CWMH were receiving at least one antimicrobial on the day of the PPS. This is similar to surveys in other LMICs reporting rates between 49 and 70% [22, 33, 34], however much higher than rates of 33–34% reported in Japan, China, Europe and an international survey [20, 35–37]. Carbapenem use at Fiji’s largest hospital was extremely low, with only one prescription recorded. In contrast carbapenems represented more than 10% of all antimicrobial prescriptions in a South American PPS [22] and 5.7% among south east Asian countries participating in a global PPS [35]. The infrequent carbapenem use in Fiji likely reflects relatively low rates of antimicrobial resistance in Pacific Island countries [38, 39] – although high-quality laboratory surveillance data is lacking – as well as CWMH’s previous stewardship efforts to restrict their use.

Assessing the appropriateness of each individual prescription was beyond the scope of this PPS. However, it is worth noting that over half of all surgical prophylaxis prescriptions continued for 2 days or longer, when one dose or 24 hours duration will usually suffice. Prolonged therapy may represent clinician concern for infection that was not reflected in the patient notes, or inappropriate extended use of antimicrobial prophylaxis. Prolonged surgical prophylaxis has been seen in recent Botswanan study, in a Europe-wide PPS, and among caesarean section patients in Ghana [33, 34, 36]. Although CWMH has an existing antimicrobial stewardship service, it currently focuses only on the highest-risk patients, such as those in ICU, or the broadest-spectrum agents. Our research has demonstrated that antimicrobials prescribed for surgical prophylaxis may represent an additional, high-yield target for stewardship interventions at CWMH.

There are some limitations of our study. Initial training provided to IPC staff was brief and no formal competency assessments were undertaken, however to compensate for this an experienced survey leader was always present in each data collecting pair. The PPS was only conducted at one hospital, which is Fiji’s largest and has a six-member IPC team, so may not reflect HAI prevalence in other hospitals in Fiji or the broader region without similar resources. As a PPS, the data presented here reflects one time-point only. Repeated surveys would provide a more reliable estimate of HAIs and antimicrobial use. No patient-level comorbidity data was collected, so we are unable to perform patient-level risk adjustment and directly compare our cohort to those from other HAI PPSs. Finally, assessment of HAIs and antimicrobial use relied heavily on medical records – there is a risk that HAIs were underestimated, or antimicrobial indications were misclassified, if documentation was poor or incomplete.

## Conclusions

We have produced the first published point prevalence survey of HAIs and AMU from the Pacific Islands region. Encouraging findings included Fiji’s relatively low overall rate of HAIs, and the low use of broad-spectrum antimicrobials. However, this study has also demonstrated important areas for improvement, such as reducing surgical site infections and the high rate of extended antimicrobials for surgical prophylaxis. While the ECDC HAI criteria appeared to perform well in our setting, other researchers in LMICs may wish to consider modification of HAI criteria if there is limited use or availability of key investigations.

## Supplementary information

**Additional file 1: Table S1.** Summary of major differences in study protocol compared to ECDC protocol. **Table S2.** Additional questions to identify patients with clinician diagnosis of HAI that failed to meet ECDC criteria.

## Data Availability

A non-identifiable copy of the dataset will be loaded onto https://bridges.monash.edu.

## Abbreviations

ABHR: Alcohol-based handrub
AMU: Antimicrobial use
CWMH: Colonial War Memorial Hospital
CVC: Central vascular catheter
ECDC: European Centre for Disease Prevention and Control
HAI: Healthcare-associated infection
ICU: Intensive care unit
IPC: Infection prevention and control
LMIC: Low- and middle-income country
LOS: Length of stay
NICU: Neonatal intensive care unit
PPS: Point prevalence survey
PVC: Peripheral vascular catheter
SSI: Skin and soft tissue infection
