# Supplementary material for: Prevalence of healthcare-associated infections and antimicrobial use among inpatients in a tertiary hospital in Fiji: a point prevalence survey
Source: Antimicrob Resist Infect Control. 2020 Aug 28;9:146. doi: 10.1186/s13756-020-00807-5 (PMC7456377; doi:10.1186/s13756-020-00807-5)
Supplement: Supplementary file 1 — Additional file 1: Table S1. Summary of major differences in study protocol compared to ECDC protocol. Table S2. Additional questions to identify patients with clinician diagnosis of HAI that failed to meet ECDC criteria. [file 13756_2020_807_MOESM1_ESM.docx]

## Additional file 1 Table S1 – Summary of major differences in study protocol compared to ECDC protocol

| **ECDC protocol** | **Deviations** | **Rationale** |  |
| --- | --- | --- | --- |
| **Data Collection Processes** | | | |
| - Composition of the team responsible for data collection varied from one hospital to another | - Data collectors included two members from the Australian research team and four local Fiji IPC staff. All local staff were trained in using the tool. Data collection occurred in two teams, each consisting of one Australian researcher, and one IPC staff member. | - To minimise the burden of time commitment required by the local IPC staff, as regular work duties were required to continue - To build capacity of local IPC staff to conduct HAI surveillance alongside experienced Australian researchers - Rotation of local IPC staff across teams helped ensure greater consistency of HAI reporting |  |
| **Patient Data Fields** | | | |
| - McCabe score was used to classify the severity of underlying medical conditions | - No risk factor data will be collected - Two “triggers” will be used for efficient data collection, as per the Australian PPS. If the patient did not have a temperature >38 and was not receiving antimicrobials or was receiving antimicrobials for a community-acquired infection, surgical or medical prophylaxis, no further data will be collected. If the patient did not fit the above criteria, then data collectors will work through the HAI algorithm. | - Insufficient resources to collect risk factor data and work through the HAI branching logic of the data collection for every patient |  |
| **Antimicrobial Usage Data Fields** | | | |
| - Anatomical Therapeutic Chemical (ATC) code noted for all antimicrobials | - No ATC code will be collected; however, the names of all antimicrobials were collected | - Reduced data collection time - ATC codes can be linked retrospectively if required |  |
| - Diagnosis (site) infection categories:  1. ASB Asymptomatic bacteriuria 2. BAC Laboratory-confirmed bacteraemia 3. BJ-O Septic arthritis, osteomyelitis, not related to surgery 4. BJ-SSI Septic arthritis, osteomyelitis of surgical site 5. BRON Acute bronchitis or exacerbations of chronic bronchitis 6. CF Cystic fibrosis 7. CNS Infections of the central nervous system 8. CSEP Clinical sepsis 9. CVS Cardiovascular infections 10. CYS Symptomatic lower urinary tract infection 11. ENT Infections of ear, nose, throat, larynx and mouth 12. EYE Endophthalmitis 13. FN Febrile neutropenia 14. GI Gastrointestinal infections 15. GUM Prostatitis, epididymo-orchitis, STD in men 16. IA Intra-abdominal sepsis, including hepatobiliary 17. OBGY Obstetric or gynaecological infections, STD in women 18. PNEU Pneumonia 19. PYE Symptomatic upper urinary tract infection 20. SIRS Systemic inflammatory response 21. SST-O Cellulitis, wound, deep soft tissue not involving bone, not related to surgery 22. SST-SSI Surgical site infection involving skin or soft tissue but not bone 23. UND Completely undefined; 24. NA Not applicable | - The following categories will be collected for each antimicrobial prescribed  1. Bloodstream 2. Bone, joint, bursa, or spinal disc 3. Cardio-vascular system 4. Central nervous system 5. Eye, ear, nose, throat or mouth 6. Gastrointestinal tract 7. Hematopoietic/Lymphoreticular 8. Reproductive tract 9. Respiratory tract 10. Skin, soft tissue or deep decubitus ulcer 11. Urinary tract 12. Other | - Reduce data collection time and simplify task for data collectors, as it is less complicated than the extensive ECDC list |  |
| **Data Validation** | | | |
| - Recommended sample size at the national level was 750 patients in 25 hospitals | - The sample size will be determined pragmatically by the number of eligible patients during the study | - This is a single site study |  |
| - Validation team was separate from the original data collection team, and recommended to be blinded | - Data collection teams, involved two international researchers and four local IPC staff, that rotated in pairs to collect data | - Not practical for this study, given single-centre and resource constraints, however the use of a data collection roster, and changing of data collection pairs, enabled greater consistency between the two teams, whilst limiting the time burden on local staff to complete their usual employment duties. - Of note, the Australiana researchers were external to CWMH |  |
| **HAI Algorithm Data Fields** | | | |
| - Surgery code list from the CDC National Healthcare Safety Network (NHSN), with a few additional codes deemed appropriate by the ECDC PPS Panel | - Removal of the below NHSN categories;  1. Abdominal aortic aneurysm repair 2. Carotid endarterectomy 3. Heart transplant 4. Kidney transplant 5. Liver transplant 6. Peripheral vascular bypass surgery 7. Refusion of spine | - Local doctors confirmed that these surgeries are never performed in CWMH. Removal of these fields helped reduce training and data collection time |  |
| - Ventilator and catheter device insertion and removal dates for diagnosed HAP and CAUTIs | - Not collected in this study | - Time constraints for data collection |  |
| - CRI-CVC Central vascular catheter-related infection & CRI-PVC Peripheral vascular catheter-related infection | - Removal of these questions in the survey | - These infections require culture from the site/device tip, investigations that are not routinely performed at CWMH. - CVC/PVC related infections are also captured in the BSI section of the survey |  |
| **HAI Details Data Fields** | | | |
| - Antimicrobial resistance phenotype highly detailed | - Sensitivities simplified to be the following options depending on pathogen:  1. For *S. aureus*: MSSA/MRSA/Unknown 2. For Enterococci: VSE/VRE/Unknown 3. For Enterobacteriaceae: Sensitive to ceftriaxone, or ceftazidime and also carbapenems/ Resistant to ceftriaxone or ceftazidime but sensitive to carbapenems/ Resistant to ceftriaxone or, ceftazidime and carbapenems 4. For Pseudomonas and Acinetobacter: Sensitive to carbapenems/ Resistant to carbapenems | - Simplified data collection - These contained AMR profiles regularly reported at CWMH - AMR susceptibility patterns at CWMH to be investigated in greater detail in subsequent research project |  |

CWMH – Colonial War Memorial Hospital. ECDC – European Centre for Disease Prevention and Control.

Table modified from Russo et al (1)

## Additional file 1 Table S2 – Additional questions to identify patients with clinician diagnosis of HAI that failed to meet ECDC criteria

| **For Hospital Acquired Pneumonia (HAP)** |
| --- |
| Data collectors proceeded through the routine survey algorithm for HAP. If no diagnosis of HAP was made, despite patient having recorded a positive response for respiratory symptoms, the following question was then asked: *“If that patient does NOT meet any of the above, was there a physician diagnosis of HAP?”* The two options were either ‘Yes’ or ‘No.’  If the selected answer was ‘Yes’, the following question would be asked: *“Were the necessary diagnostic tests performed to satisfy criteria for this HAI?”* The five checkbox options were: ‘Necessary Microbiology not performed’, ‘Necessary Radiology not performed’, ‘Necessary Microbiology performed, but negative’, ‘Necessary Radiology performed, but negative’ and ‘Patient did not satisfy WBC or fever criteria.’  Patients recorded as having ‘Necessary Microbiology not performed’ or ‘Necessary Radiology not performed’ were counted as potential additional HAP cases that had failed to meet ECDC criteria. If all relevant investigations had been performed but were negative, or patients failed to satisfy WBC or fever criteria, these patients were not considered to be potential additional HAP cases. |
| **For Skin/Soft Tissue infection (SSTI)** |
| Data collectors proceeded through the routine survey algorithm for SSTI. If no diagnosis of SSTI was made, despite patient having recorded a positive response for skin/soft tissue symptoms, the following question was then asked: *“If that patient does NOT meet any of the above, was there a physician diagnosis of skin, decubitus ulcer or soft tissue infection?”* The two options were either ‘Yes’ or ‘No.’  If the selected answer was ‘Yes’, the following question would be asked: *“Were the necessary diagnostic tests performed to satisfy criteria for this HAI?”* The four checkbox options were: ‘Necessary Microbiology not performed’, ‘Necessary Radiology not performed’, ‘Necessary Microbiology performed, but negative’, and ‘Necessary Radiology performed, but negative.’  Patients recorded as having ‘Necessary Microbiology not performed’ or ‘Necessary Radiology not performed’ were counted as potential additional SSTI cases that had failed to meet ECDC criteria. If all relevant investigations had been performed but were negative, these patients were not considered to be potential additional SSTI cases. |
| **For *C. difficile* infection** |
| As CWMH does not perform any laboratory testing for *C. difficile*, it was anticipated that there would be no *C. difficile* HAI cases meeting ECDC criteria.  Following the ECDC standard questions on *C. difficile*, the following question would be asked:  *“Was there a physician diagnosis of C. difficile? AND are one of the following true:*  *- Onset of symptoms occurred on Day 3 or later in current admission*  *- Patient was discharged from an acute care hospital in the preceding 28 days from onset of symptoms”*  The two options were either ‘Yes’ or ‘No.’  If the selected answer was ‘Yes’, the following question would be asked: *“Were the necessary diagnostic tests performed to satisfy criteria for this HAI?”* The four checkbox options were: ‘Necessary Microbiology not performed’, ‘Necessary Radiology not performed’, ‘Necessary Microbiology performed, but negative’, and ‘Necessary Radiology performed, but negative.’  Patients recorded as having ‘Necessary Microbiology not performed’ or ‘Necessary Radiology not performed’ were counted as potential additional *C. difficile* HAI cases that had failed to meet ECDC criteria. |

CWMH – Colonial War Memorial Hospital. ECDC – European Centre for Disease Prevention and Control.

**References**

1. Russo PL, Stewardson AJ, Cheng AC, Bucknall T, Mitchell BG. The prevalence of healthcare associated infections among adult inpatients at nineteen large Australian acute-care public hospitals: a point prevalence survey. Antimicrobial resistance and infection control. 2019;8:114.
